# Cancer risk in immune-mediated inflammatory diseases (IMID)

**DOI:** 10.1186/1476-4598-12-98

**Published:** 2013-08-29

**Authors:** Rudi Beyaert, Laurent Beaugerie, Gert Van Assche, Lieve Brochez, Jean-Christophe Renauld, Manuelle Viguier, Veronique Cocquyt, Guy Jerusalem, Jean-Pascal Machiels, Hans Prenen, Pierre Masson, Edouard Louis, Filip De Keyser

**Affiliations:** 1 Department of Biomedical Molecular Biology, Ghent University, VIB, Ghent, Belgium; 2 Department of Molecular Biomedical Research, Ghent University, VIB, Ghent, Belgium; 3 Department of Gastroenterology, AP-HP, Hôpital Saint-Antoine, Paris F-75012, France; 4 UPMC Univ Paris, Paris 06 F-75005, France; 5 Department of Gastroenterology, University Hospitals Leuven, Leuven, Belgium; 6 Department of Dermatology, Ghent University Hospital, Ghent, Belgium; 7 Ludwig Institute for Cancer Research and Experimental Medicine Unit, Université Catholique de Louvain, Brussels, Belgium; 8 Assistance-Publique-Hôpitaux de Paris, Paris, France; 9 Dermatology department, Université Paris VII Denis-Diderot, Hôpital Saint-Louis, Paris, France; 10 Department of Medical Oncology, Ghent University Hospital, Ghent, Belgium; 11 Department of Medical Oncology, University of Liège, Liège, Belgium; 12 Department of Medical Oncology Unit, Université Catholique de Louvain, Brussels, Belgium; 13 Cliniques Universitaires Saint-Luc, Brussels, Belgium; 14 Department of Digestive Oncology, University Hospital Gasthuisberg, Leuven, Belgium; 15 de Duve Institute, Université Catholique de Louvain, Brussels, Belgium; 16 Gastroenterology, CHU and University of Liege, Liège, Belgium; 17 Department of Rheumatology, Ghent University, 0K12, De Pintelaan 185, Ghent B-9000, Belgium

**Keywords:** Antirheumatic agents, Autoimmune diseases, Biological products, Cancer, Inflammation, Tumor necrosis factor

## Abstract

Inflammation and cancer have a profound yet ambiguous relationship. Inflammation - especially chronic inflammation - has protumorigenic effects, but inflammatory cells also mediate an immune response against the tumor and immunosuppression is known to increase the risk for certain tumors.

This article reviews current literature on the role of inflammation in cancer and the cancer risk in immune-mediated inflammatory diseases (IMIDs). We discuss the effect on cancer risk of different drug classes used in the treatment of IMIDs treatment, including biologicals such as tumor necrosis factor (TNF) inhibitors.

Overall cancer incidence and mortality risk are similar to the general population in inflammatory bowel disease (IBD), and slightly increased for rheumatoid arthritis and psoriasis, with risk profiles differing for different tumor types. Increased risk for non-melanoma skin cancer is associated with thiopurine treatment in IBD, with the combination of anti-TNF and methotrexate in rheumatoid arthritis and with PUVA, cyclosporine and anti-TNF treatment in psoriasis. Data on the safety of using biologic or immunosuppressant therapy in IMID patients with a history of cancer are scarce.

This review provides clinicians with a solid background to help them in making decisions about treatment of immune-mediated diseases in patients with a tumor history.

This article is related to another review article in Molecular Cancer: http://www.molecular-cancer.com/content/12/1/86.

## Introduction

Immune-mediated inflammatory diseases (IMIDs) are a group of chronic and highly disabling diseases involving inappropriate or excessive immune responses caused or accompanied by cytokine dysregulation and acute or chronic inflammation [1]. This includes a wide variety of illnesses, such as Crohn’s disease (CD), ulcerative colitis (UC), psoriasis, rheumatoid arthritis (RA), and systemic lupus erythematosus (SLE). IMIDs are fairly common, affecting an estimated 5% to 7% of the population in Western countries. Treatment of IMIDs focuses on the rapid control of inflammation, prevention of tissue damage, and where possible, long-term remission of the disease. This is achieved using corticosteroids, immunosuppressants, and “biologicals”, especially those targeting tumor necrosis factor (TNF).

Because immunosurveillance is thought to help suppress the development of cancer, there are concerns that immunotherapies might increase cancer risk in patients with IMIDs. Furthermore, inflammation is known to have both pro- and anti-tumorigenic effects, and cancer-related inflammation is now considered the seventh hallmark of cancer [2].

In this article, we discuss the current state of knowledge concerning the relationship between IMIDs and cancer. This information should help clinicians decide about the safety of giving immunosuppressive therapies to IMID patients with a history of tumors.

## Dual relationship between inflammation and cancer

Inflammation can occur in response to dietary or environmental factors, infection, and autoimmune diseases including IMIDs. Inflammatory cells are present in most, if not all, solid tumors [3]. Tumor-associated macrophages can comprise up to as much as half of the mass of a solid tumor [4]. These cells promote tumor cell survival, proliferation, and dissemination, and a high level of them is associated with a poor prognosis. Tumor-associated inflammatory cells appear to be actively recruited, possibly as part of an anti-tumor response, but this inflammatory response may be usurped by the tumor to promote tumorigenesis [3].

### Anti-tumorigenic effects of inflammation

Activation of inflammatory cells as part of an immune response to eliminate mutant cells, a process called immunosurveillance, was originally suggested by Ehrlich and later formalized by Burnet and Thomas [5]. Aberrant proteins or peptide-MHC complexes on the surfaces of transformed or malignant cells are recognized and targeted for elimination by the immune system. Evidence that the immune system recognizes and eliminates tumor cells was originally obtained in mice, but this is also supported by several lines of clinical evidence: cancer incidence is increased in transplant patients treated with immunosuppressants; cancer patients develop immune responses to tumors; immune responses in tumors correlate with improved prognosis in colorectal cancer; survival positively correlates with the presence of tumor-infiltrating lymphocytes, CD8^+^ T cells, and natural killer cells in various cancers; higher natural cytotoxic activity of peripheral blood lymphocytes correlates with a reduced cancer risk [6-8].

Murine studies found that tumors formed in the absence of an intact immune system are more immunogenic in wild-type mice than those formed in the presence of an intact immune system [9-13]. In other words, the immune response eliminated the more immunogenic cells and selected less immunogenic cells, a process that Dunn and colleagues refer to as “immunologic sculpting” or “immunoediting” [7]. Immunoediting is composed of three phases: “elimination”, “equilibrium”, and “escape”. The initial phase of an immune response to a tumor, elimination, is the same as immunosurveillance and results in destruction of (part of) the tumor cells [7]. Dunn and colleagues envision that the tumor thereafter remains in equilibrium with the immune system, wherein selection pressure continues but is unable to eliminate the tumor. In this equilibrium phase, some tumor cells are eliminated and others, including new variants, survive. In the final escape phase, the selected tumor cell variants have become resistant to elimination by the immune system.

Efficient inhibition of tumor growth was recently shown to involve not only defined cell death and clearance mechanisms by CD8+ cytotoxic T lymphocytes and natural killer cells, but also the induction of tumor cell senescence by interferon-γ and TNF producing CD4+ T-helper 1 cells. In addition, T-helper 1 immunity can also induce anti-angiogenic chemokines that protect against cancer [14].

### Pro-tumorigenic effects of inflammation

A wide range of studies indicate that inflammation also contributes more directly to tumorigenesis [15]. Nearly one in five cancers is linked to infections: e.g. Helicobacter pylori and Hepatitis C virus infections eventually lead to gastric and liver cancers, respectively [16,17]. Also, many cancers are associated with persistent inflammation due to environmental factors or autoimmune reactions: e.g. lung cancer is associated with asbestosis and smoking, colon cancer with inflammatory bowel disease (IBD), and lymphoma with celiac disease [8,17]. Furthermore, numerous experimental and epidemiologic studies, along with randomized clinical trials showed that long-term daily use of the nonsteroidal anti-inflammatory drug aspirin reduced the incidence of several cancers, especially those of the gastrointestinal tract [18-21]. The mechanism of action of the chemopreventive and anticancer effects of aspirin is not fully understood, but it has been attributed to its anti-inflammatory effects, specifically inhibition of prostaglandin-endoperoxide synthase 2 (formerly named cyclooxygenase 2), which is the rate-limiting step for the conversion of arachidonic acid to prostaglandins. Because aspirin can cause stomach upset and dangerous internal bleeding, its use as anticancer drug for the general population is still under debate [22]. Together, these findings suggest that when inflammation becomes persistent or dysfunctional, it can promote tumor growth, as inflammatory cells, normally recruited to control damage, are diverted by the tumor for pro-tumorigenic purposes [3,23].

How chronic inflammation increases cancer risk is beginning to come into focus [3,15]. Chronic inflammation can initiate tumors by directly causing DNA changes or making cells more susceptible to mutagens. In addition, inflammation can act as a tumor promoter. Inflammatory mediators, including cytokines like TNF, interleukin (IL)-1, and IL-6, growth factors, chemokines, and proteases produced by tumor-associated lymphocytes and macrophages can enhance tumor cell growth and metastasis by promoting their survival, proliferation, migration to and invasion of other tissues. Tumor-associated macrophages release inflammatory mediators that stimulate tumor angiogenesis and lymphangiogenesis [4,23], and produce cytokines, including transforming growth factor (TGF) β and IL-10, that can directly suppress immune responses [24]. Also, myeloid-derived suppressive cells, which accumulate in infections, inflammatory and autoimmune diseases, and cancer, can inhibit tumor immunosurveillance and suppress natural killer cells [25].

At the molecular level, the transcription factor NF-κB appears to be a key connecting element between inflammation and cancer [26]. NF-κB is a central intracellular transducer of inflammatory signals [27], integrating signals from a variety of environmental changes, including infection, tissue damage and autoimmunity [26]. Several proinflammatory cytokines such as TNF and IL-1 are potent activators of NF-κB, which regulates the transcription of a variety of inflammatory genes, including TNF and IL-1 themselves, thus further amplifying the proinflammatory signal [26-28]. Not surprisingly, NF-κB is activated in many inflammatory diseases, and inhibitors of NF-κB have beneficial effects in mouse models of inflammation [29]. Additionally, mutant forms of NF-κB, NF-κB inhibitor proteins such as IκBα and A20, or upstream signaling components that feed into NF-κB, are found in many cancers [30]. NF-κB activation is thought to act as a tumor promoter by enhancing tumor cell survival and proliferation and helps convert tumor-associated macrophages to a tumor-promoting phenotype [26]. Despite these pro-oncogenic roles, drugs specifically targeting NF-κB have had limited success in treating cancer, although ubiquitin-proteasome targeting drugs such as bortezomib and carfilzomib, which act in part by preventing NF-κB activation, have been successfully approved for clinical application while some other promising candidates are currently under clinical trials [31].

NF-κB also regulates the expression of IL-6, a multifunctional cytokine that plays important roles in immune responses, cell survival and proliferation. Mouse studies demonstrated that IL-6 is important for both tumor development and growth in colitis associated cancer, with IL-6 promoting both proliferation and survival of intestinal epithelial cells via the activation of the transcription factor STAT3 [32,33]. Although in many cancers STAT3 is not directly activated by oncogenic mutations, it exerts critical oncogenic functions in both cancer and immune cells within the microenvironment [34]. More recently, mouse studies also demonstrated a critical role for IL-23 and its downstream cytokines IL-17 and IL-22 in the development of colitis-associated cancer [35], increasing the number of cytokines that link inflammation with the development of cancer.

## IMIDs and Cancer Risk

IMIDs are characterized by severe inflammation [1]. Given the molecular and cellular links between inflammation and cancer, it is not surprising that many IMIDs are associated with an increased risk of cancer (Table 1), although confounding effects of treatment are hard to eliminate.

**Table 1:** Examples of IMIDs associated with increased risks for cancer

**IMID**	**Associated malignancies**
Aplastic anemia	Myeloproliferative disorders [36]
Autoimmune hepatitis	Non-melanoma skin cancer, hepatocellular carcinoma [37,38]
Celiac disease	Non-Hodgkin’s lymphoma, esophageal cancer, Hodgkin’s lymphoma, small bowel carcinoma, stomach cancer [39-41]
Crohn’s disease	Colorectal and fistula cancer and for cancer of the small bowel, upper gastrointestinal tract, lung, urinary bladder and skin [42-44]. Liver, pancreatic, prostate, testicular, and kidney cancers, nonthyroid endocrine tumors, and leukemia [45].
Dermatomyositis	Ovarian, lung, gastric cancer [46]
Giant cell arteritis	Myeloproliferative disorders [36]
Immune thrombocytopenic purpura	Myeloproliferative disorders [36]
Polymyalgia rheumatica	Myeloproliferative disorders [36]
Primary biliary cirrhosis	Hepatocellular carcinoma [38]
Psoriasis	Colon cancer [47] and possibly cancer of the urinary bladder, kidney, oropharynx/larynx, esophagus, stomach, liver/gallbladder, vulva, female breast, and pancreas and for leukemia, non-Hodgkin’s lymphoma, and non-melanoma skin cancer [48-57]
Reiter’s syndrome	Myeloproliferative disorders [36]
RA	Lymphoma and leukemia, non-melanoma skin cancers [32,33,58]; lymphoma and lung cancer [33]
Sarcoidosis	Rectal, colon, kidney, skin (squamous cell), nonthyroid endocrine cancer; non-Hodgkin’s lymphoma; leukemia [59]
Sjögren syndrome	lymphoproliferative disorders [46]
SLE	Hematological malignancies, including non-Hodgkin’s lymphoma, and cancers of the vagina/vulva/cervix, nasopharynx, and kidney [60-62]
Systemic sclerosis	Lung, skin, esophageal, non-melanoma skin, and liver cancer [40,46,63]
Type 1 diabetes mellitus	Pancreatic cancer [64,65]
Ulcerative colitis	Colorectal carcinoma, liver-biliary cancer, and leukemia [42,66]

### Rheumatoid arthritis

Although its triggers are unknown, RA is considered the archetypal IMID in which autoimmunity induces the production of the pro-inflammatory cytokines TNF, IL-1, and IL-6, triggering the production of degradative enzymes that destroy the joints and further stimulate the T cell response [1].

Two Swedish population-based studies found that patients with RA are at an approximately two-fold increased risk for lymphoma and leukemia, a 20% to 50% increased risk for respiratory tract cancer, and a 70% increased risk for non-melanoma skin cancers (NMSC), but at decreased risk for breast and colorectal cancer [30,32]. A meta-analysis including 21 publications confirmed the increased risk for lymphoma and lung cancer and decreased risk for colorectal and breast cancer in RA [33]. Increased lymphoma risk is limited, however, to the subset of RA patients with longstanding and very severe disease [34].

### Inflammatory bowel diseases

Crohn’s disease and ulcerative colitis represent the two principal forms of IBD [35]. Both are chronic inflammatory diseases apparently caused by an inappropriate immune response, probably to a gut antigen that is normally suppressed [1]. Both diseases are characterized by severe gastrointestinal inflammation, although they also have systemic manifestations affecting the skin, eyes, joints, liver, hepatobiliary system [1,67].

Overall cancer incidence rates are increased in CD, but similar to the general population in UC [68]. Meta-analyses indicate that patients with CD are at increased risk for colorectal and fistula cancer and for cancer of the small bowel, upper gastrointestinal tract, lung, bladder and skin [42-44]. A Swedish registry study of more than 27,000 CD patients hospitalized between 1964 and 2004 found elevated risks for liver, pancreatic, prostate, testicular, and kidney cancers, nonthyroid endocrine tumors, and leukemia [45].

Patients with UC have an increased risk of colorectal carcinoma, liver-biliary cancer, and leukemia but a reduced risk of pulmonary cancer [42,66]. The risk of colorectal cancer in UC is further elevated in patients with dysplasia and ongoing mucosal inflammation [69-71].

### Psoriasis

Psoriasis is a chronic inflammatory skin disease characterized by circumscribed, erythemato-squamous plaques with adherent scales [72]. Psoriasis appears to be driven by a dysregulation of the innate immune system mediated by IFN-α, although several other inflammatory mediators, including TNF, are also involved. Psoriasis may be associated with systemic manifestations, including an increased risk for metabolic syndrome, cardiovascular disease and systemic inflammation similar to that observed in RA.

Several studies from the pre-biologics era implicated an increased risk for lymphoma, NMSC and cancers related to alcohol and smoking in psoriasis patients [48]. Cancer risk seems to be higher in patients with severe psoriasis, which raises the question whether this is caused by chronic inflammation or by the systemic treatments more often used in severe psoriasis [49,73].

The Iowa Women’s Health Study, which included more than 32,000 women, found a significant association only between colon cancer and psoriasis when disease incidence was adjusted for smoking, body mass index, education, physical therapy, and use of hormone therapy [47]. Other studies suggest an increased risk for cancer of the bladder, kidney, oropharynx/larynx, esophagus, stomach, liver/gallbladder, vulva, breast, and pancreas and for leukemia, non-Hodgkin’s lymphoma, and NMSC [48-55]; however, these studies did not control for environmental factors such as alcohol and smoking. Although concern has been raised that psoriasis treatment with PUVA (psoralen + ultraviolet light), methotrexate, or cyclosporine can increase cancer risk [74,75]; the most recent studies have shown that these treatments are not associated with an increased risk [50,76,77].

Very recently, a systematic literature review with meta-analysis was performed on the risk of cancer in psoriasis [56], accompanied by evidence-based recommendations [57]. Together, the authors concluded that there is a slightly increased risk of some cancers in patients with psoriasis (upper aero-digestive tract, liver, lung, pancreatic and urinary tract cancers), that the highest increased risk is for skin carcinoma, that there is no increased risk of melanoma and that regarding lymphoma, misdiagnosis of primary skin lymphoma as psoriasis might have overestimated the risk.

### Systemic Lupus Erythematosus (SLE)

SLE is another chronic inflammatory disease triggered by an autoimmune reaction and mediated by inflammatory cytokines, especially TNF, IL-1 and type 1 interferons [78]. SLE can affect almost any tissue and is most often characterized by fatigue coupled with musculoskeletal, skin, pulmonary, cardiac, gastrointestinal, renal, neuropsychiatric, or reproductive manifestations [79]. SLE is associated with an increased risk of hematological malignancies, including non-Hodgkin’s lymphoma, and cancers of the vagina/vulva/cervix, nasopharynx, and kidney [60-62], but a decreased risk of breast, ovarian, and endometrial cancer [80].

## IMID treatments and cancer risk

### Risk of cancer associated with immune suppression: transplantation as a model

Treatment of IMIDs focuses on inhibiting inflammation by suppressing the activity and proliferation of immune cells and the cytokine production involved in innate and adaptive immune responses [81]. Before the advent of biologics, this could only be accomplished with immunosuppressant drugs. However, experience in transplant patients indicates that these drugs increase the risk of skin cancer, especially NMSC, and the risk of Epstein-Barr virus-associated post-transplant lymphoproliferative disorder [82]. This effect of immunosuppressants provides support for the immunosurveillance hypothesis [5,83,84].

### Immune modulators

#### Thiopurines

The thiopurines azathioprine, 6-thioguanine, and 6-mercaptopurine are immunosuppressive drugs commonly used for the treatment of autoimmune diseases, for the prevention of transplant rejection and the treatment of lymphoproliferative disorders [85]. Thiopurines act in several ways to interfere with lymphocyte proliferation. Accumulating evidence shows that they increase the cancer risk in IMID patients. IBD patients treated with thiopurines have an increased risk for NMSC and lymphoma [86,87].

A 2005 meta-analysis of cohort studies found that treatment of IBD with azathioprine or 6-MP increased the risk of lymphoma (RR = 4.2), although it was not clear whether the increased risk was due to the medications, the severity of the underlying disease, or a combination of both [88]. This conclusion was supported by a French observational study of 19,486 IBD patients followed up for a median of 35 months, which found an increased risk of lymphoproliferative disorders in patients receiving thiopurines compared to those who had never received them (HR = 5.28) [86]. In addition, two retrospective analyses reported an increase in NMSCs associated with IBD treatment (adjusted OR = 4.3 and OR = 5.0, respectively) [87,89].

#### Cyclosporine

Cyclosporine is a calcineurin inhibitor that inhibits inflammation by blocking IL-2 production by activated CD4^+^ T cells [75]. In transplant patients, cyclosporine increases the risk of lymphoma, internal malignancies, and skin cancers [75]. A 2003 report found that in patients with psoriasis, cyclosporine increases the risk of skin cancer 6-fold [90]. The risk increased with treatment duration over 2 years and prior phototherapy. A more recent systematic review found that cyclosporine significantly increases the risk for NMSC in RA [91].

#### Methotrexate

Methotrexate is a folic acid analog that suppresses cell proliferation by inhibiting DNA synthesis [75]. Melanoma and Epstein-Barr virus-associated lymphomas in psoriasis patients taking methotrexate have been reported [92]. However, a Canadian observational study including 23,810 patients with RA did not find an increased risk for hematologic malignancies in patients treated with methotrexate [92]. This was also found in a 2009 meta-analysis by Salliot and van der Heijde, which reported no increase in the risk of lymphoma or malignancies in RA patients treated with methotrexate [93]. However, a 2010 systematic review by Krathen et al. suggested that methotrexate in RA may increase the risk of malignant melanoma, and double the risk for NMSC when combined with anti-TNFs; in patients with psoriasis methotrexate increased the risk of NMSC [94].

#### Cyclophosphamide

Cyclophosphamide suppresses immune function by inhibiting lymphocyte proliferation [95]. A Canadian observational study including 23,810 RA patients reported an increased risk of hematologic malignancies (unadjusted OR = 2.21) in patients treated with cyclophosphamide [92]. Cyclophosphamide is known to increase bladder cancer risk, and patients who have received this drug should be monitored regularly for microscopic hematuria [96].

### Biologics Targeting TNF

TNF, as its name implies, was discovered as a serum factor from lipopolysaccharide-treated mice that causes tumor necrosis [97], and was originally intensively studied because of its potential use as anti-tumor agent [98]. However, initial enthusiasm about its clinical use as anti-tumor agent was curbed due to significant toxicities and lack of efficacy of systemic treatment. Clinical use of TNF for cancer treatment is therefore limited to the setting of hyperthermic isolated limb perfusion for the regional treatment of locally advanced soft tissue sarcomas, metastatic melanomas and other irresectable tumors to avoid limb amputation [99]. Moreover, a paradoxical tumor-promoting role of TNF became apparent, which may reflect the role of TNF as a key pro-inflammatory mediator and the tumor-promoting role of inflammation [100]. TNF is a key mediator of inflammation, which is barely detectable in circulation under normal conditions, but is produced by macrophages, activated T cells, natural killer cells, mast cells, and stromal cells during innate or adaptive immune responses [101,102].

TNF acts on multiple cell types by binding to specific cell surface receptors that activate multiple signaling pathways that culminate in the activation of MAP kinase, NF-κB and other transcription factors. At high doses, TNF causes the death of tumor blood vessels, although at lower doses, it can act as a tumor promoter and enhancer of metastasis [16]. Furthermore, certain polymorphisms in the TNF gene are associated with hepatocellular cancer [103-106], non-Hodgkin’s lymphoma [107], breast cancer [108], and gastric cancer [109].

TNF is an important mediator of the dysregulated immune and inflammatory function in IMIDs. Drugs that target TNF are the most widely used biologics for treating IMIDs, with five drugs currently approved for clinical treatment of IMIDs (Table 2) [110]. Although anti-TNF therapy is used in different IMIDs, not all anti-TNF agents have the same efficacy in all IMIDs [111].

**Table 2:** Biologics used in the treatment of IMIDs

**Biologic**	**Type of molecule**	**IMIDs for which it is licensed**
Infliximab (Remicade®)	Chimeric monoclonal antibody against TNF	RA, psoriatic arthritis, ankylosing spondylitis, ulcerative colitis, Crohn’s disease, plaque psoriasis
Adalimumab (Humira®)	Human monoclonal antibody against TNF	RA, psoriatic arthritis, ankylosing spondylitis, Crohn’s disease, ulcerative colitis, plaque psoriasis, polyarticular juvenile idiopathic arthritis
Golimumab (Simponi®)	Human monoclonal antibody against TNF	RA, psoriatic arthritis, ankylosing spondylitis
Certolizumab pegol (Cimzia®)	Pegylated humanized Fab’ monoclonal antibody fragment against TNF	RA, Crohn’s disease
Etanercept (Enbrel®)	Fusion protein of two TNF receptor 2 extracellular domains and the Fc portion of human IgG	RA, polyarticular juvenile idiopathic arthritis, psoriatic arthritis, ankylosing spondylitis, plaque psoriasis
Tocilizumab (Actemra® or RoActemra®)	Humanized monoclonal antibody against the interleukin-6 receptor	Juvenile idiopathic arthritis, RA
Rituximab (Rituxan® or MabThera®)	Chimeric anti-CD20 monoclonal antibody	RA, granulomatosis with polyangiitis (Wegener)
Abatacept (Orencia®)	Fusion of extracellular domain of human CTLA-4 and the Fc domain of IgG	RA
Alefacept (Amevive®)	Fusion of CD2-binding region of LFA-3 and the CH2 and CH3 domains of IgG1	Psoriasis
Anakinra (Kineret®)	Recombinant, non-glycosylated human IL-1 receptor antagonist	RA

Studies on the effect of TNF-blockers on the incidence of malignancy began to appear in the late 1990s. Bickston et al. reported the occurrence of lymphoma after infliximab treatment in patients with CD in 1999 [112]. Other individual reports of lymphoproliferative diseases in patients treated with infliximab or etanercept followed soon thereafter [113-115]. A large prospective observational study including 18,572 patients from the US National Data Bank for Rheumatic Diseases found a 2.9-fold increase in the incidence of lymphoma in patients treated with etanercept or infliximab [116]. Updates three years later from this same database found the contrary – that infliximab and etanercept, after a median exposure of three years, were not associated with a risk of lymphoma in patients with RA [117,118]. Two additional observational studies reported that anti-TNF-biologics do not increase the risk of solid cancer, lymphoma or leukemia [30,32].

Thus, while some of the studies suggested a link between the use of anti-TNF biologics and cancer, others did not. Interpretation and reconciliation of these different findings is difficult because randomized clinical trials are usually too small or brief to obtain sufficient data to accurately determine the effects of a treatment on cancer and because most observational studies usually lack adequate control groups. Meta-analyses have therefore been performed on pooled data from multiple randomized clinical studies or prospective observational studies to better assess the risks of cancer associated with the treatment of IMIDs by biologics (Table 3).

**Table 3:** Meta-analyses on cancer risks associated with the use of biologics to treat IMIDs

**Study**	**Disease**	**Study types included**	**Medication**	**Findings**
Bongartz et al. 2006 [119]	RA	Prosp Obs	Infliximab Adalimumab	Increased risk of malignancies
Peyrin-Biroulet et al. 2008 [120]	Crohn’s disease	RCTs	Infliximab, adalimumab, certolizumab, CDP571	No increase in malignancy
Leombruno et al. 2009 [121]	RA	RCTs	Etanercept, infliximab, adalimumab	No increased risk of melanoma, lymphoma, non-lymphoma skin cancer, or cutaneous cancer + melanoma
Bongartz et al. 2009 [122]	RA	RCTs	Etanercept for ≥ 12 weeks	Non-significant increase in cancer
Siegel et al. 2009 [123]	Adult Crohn’s disease	RCTs	Infliximab, adalimumab, certolizumab	Increase risk of non-Hodgkin’s lymphoma
Mariette et al. 2011 [124]	RA	Prosp Obs	Anti-TNF	No increase in malignancy (including lymphoma). Increase in skin cancer (including melanoma)
Dommasch et al. 2011 [125]	Plaque psoriasis, psoriatic arthritis	RCTs	Etanercept, Infliximab, adalimumab, certolizumab, golimumab	No increase in cancers for short-term use
Askling et al. 2011 [126]	Any	RCTs	etanercept, infliximab, adalimumab	No increase in cancer with short-term use
Campbell et al. [127]	RA	RCTs	Tocilizumab	No increase in risk of malignancy
Lopez-Olivo et al. [128]	RA	RCTs	Adalimumab, Certolizumab, Etanercept, Golimumab, Infliximab, Abatacept, Anakinra, Rituximab, Tocilizumab	No increased risk of malignancy in comparison with DMARDs or placebo
Moulis et al. 2012 [129]	RA	RCTs	Anti-TNF	No excess cancer risk in either per protocol or intention to treat analysis, non-significant trend for increased non-melanoma skin cancer

Abbreviations: *DMARDs* Disease-modifying anti-rheumatic drugs, *IMID* immune-mediated inflammatory disease, *RCT* randomized clinical trial, *Prosp Obs* prospective observational trial.

An initial meta-analysis published in 2006 by Bongartz et al. [119] reported a 3.3-fold dose-dependent increase in malignancies associated with infliximab in RA patients. A 2011 meta-analysis by Mariette et al. [124] analyzing data from 21 prospective observational studies on anti-TNF biologics in RA found that although anti-TNF biologics are not associated with an increase in malignancies, especially lymphoma, they are associated with an increase in the risk of skin cancer, including melanoma.

Meta-analyses of randomized clinical trials, however, found that the treatment of RA with anti-TNF biologics [121,126,129] or biologics overall [128] does not significantly increase the risk for any type of cancer in RA patients An important limitation of the RCT data in these studies, however, is the shortness of the follow-up period of the included studies in comparison with the latency period for emergence of cancer.

An integrated analysis of three patient databases found that the risk of malignancy in RA patients treated with anti-TNF biologics does not increase with time [130].

The risk for cancer for individual anti-TNF biologics other than infliximab has been assessed in a few studies. An observational in 2004 found an increased risk of lymphoma associated with etanercept therapy in RA [116], this was not supported by their 2007 updates from the same observational database, although they did find an increased risk of skin cancer [117,118]. A meta-analysis of randomized clinical trials from 2009 found that the use of etanercept for 12 weeks or more in patients with RA was associated with a non-significant increase in the incidence of cancer [122]. Also, a 2010 report found that the use of golimumab at the FDA-approved dose to treat RA was not associated with a difference in the rate of cancer rate [131].

A prospective observational study in France by Mariette et al. [132] found that patients receiving adalimumab or infliximab have a higher risk for lymphoma than those treated with etanercept and that exposure to adalimumab or infliximab versus etanercept was an independent risk factor for lymphoma. The 2011 meta-analysis by Askling et al. [126] did not find differences between the anti-TNF antibodies adalimumab, etanercept, and infliximab, although they suggest that this may have been due to differences in statistical precision or in baseline cancer risk between the various studies.

Few studies have examined the risk of cancer associated with the use of anti-TNF biologics in IMIDs other than RA. A 2008 meta-analysis of randomized clinical trials by Peyrin-Biroulet et al. reported that the treatment of CD by anti-TNF biologics does not increase the risk for cancer [120]. However, a more recent meta-analysis of randomized clinical trials by Siegel et al. found that treatment of CD with anti-TNF biologics in combination with immunomodulators is associated with an increased risk of non-Hodgkin’s lymphoma [123]. Analysis of data from the FDA Adverse Event Reporting System (AERS) showed that treatment with a combination of thiopurines and TNF inhibitors, but not with TNF inhibitors alone is associated with increased risk of non-Hodgkin lymphoma in IBD patients [133]. Also, a meta-analysis of randomized clinical trials in plaque psoriasis and psoriatic arthritis found that short-term anti-TNF biologic use is not associated with a significant increased risk of cancer [125].

### Biologics targeting molecules other than TNF

#### Alefacept

Alefacept is a human fusion protein of the CD2-binding region of LFA-3 and the CH2 and CH3 domains of IgG1. It inhibits T cell activation and induces apoptosis of memory T cells and has been approved for the treatment of psoriasis [134]. Very little information is available on the risk of cancer with alefacept, although an integrated analysis of data from 13 clinical trials including 1869 patients found that this agent does not cause a dose-dependent increase in the incidence of malignancy [135].

#### Rituximab

Rituximab is a chimeric monoclonal antibody against the protein CD20 that induces B cell killing and has been approved for the treatment of RA refractory to anti-TNF treatment [136]. Few data are available on whether rituximab use in RA patients affects the risk of cancer, although a recent analysis of 186 patients followed for an average of 22 months found no increase in the overall cancer rate compared to patients treated with disease-modifying anti-rheumatic drugs (DMARDs) [137].

C Tarella et al. [138] described the long-term outcome of a large series of patients with lymphoma who received an intensive chemotherapy schedule, with or without addition of four to six doses of rituximab. In this study, rituximab addition was associated with an increased risk of solid tumor occurrence both in univariate and multivariate analysis. Conversely, rituximab had a significant protective role on the risk of death. This finding in lymphoma patients is noteworthy and should be further explored. Caution is therefore also warranted in other disease backgrounds. The different patient background and the combination of rituximab with particular anticancer drugs in the lymphoma population, however, precludes simple extrapolation of the data from the lymphoma population to the IMID field.

#### Abatacept

Abatacept is a fusion protein of IgG and the extracellular domain of CTLA-4 that inhibits T cell activation approved for treatment of moderate to severe RA [139]. An integrated analysis of five registries comprising 4134 RA patients found similar overall rates of cancer in patients treated with abatacept as in the general RA population [140]. A long-term extension of an RCT with a five-year follow-up period, showed no increased malignancy rates under abatacept treatment [141].

#### Ustekinumab

Ustekinumab is a blocking antibody to the IL-12/IL-23 receptor approved for the treatment of plaque psoriasis [142]. Cumulative safety data from four phase II and III studies including 3117 patients shows no increase in malignancy rate during the first four years of ustekinumab treatment [143].

#### Anakinra

Anakinra is a competitive inhibitor of IL-1 binding to the IL-1 receptor approved for the treatment of RA [144]. Prospective observational data from the German database RABBIT demonstrated no increase in the overall cancer rate in RA patients treated with anakinra [145].

#### Tocilizumab

Tocilizumab is a humanized mouse monoclonal antibody to the IL-6 receptor that blocks ligand binding [146][133]. A 2010 meta-analysis and systematic review of randomized clinical trials found that tocilizumab does not significantly increase the rate of malignancies [127].

### Biologics in patients with cancer history

A 2010 study of data from the British Society for Rheumatology Biologics Register reported that in patients with RA, prior malignancy and an average of three years of follow up, the malignancy incidence rate did not increase in patients receiving anti-TNF therapy [147]. The authors concluded that the way in which the patients were selected for anti-TNF therapy is not leading to an increased risk of incident malignancy but cautioned against concluding that it is safe to use anti-TNF biologics for RA patients with prior malignancy.

Up to now there is no clear evidence to guide the decision whether or not to use immunosuppressants or biologics in patients with previous history of cancer. Treatment decisions in these patients must taken on a case-by-case basis after discussion with a multidisciplinary team, taking into account the need for such treatment, the risk of cancer recurrence and the sensitivity of a specific cancer to immunosuppression, finally weighing benefits versus risks for the patient.

## Conclusion

The evidence provided in this manuscript leads to the conclusion that some IMIDs are associated with an increased cancer risk and that the cancer risk associated with immunotherapy should be discussed in relation with each individual conventional or biological drug.

Overall cancer incidence and mortality risk are similar to the general population in IBD, and slightly increased for RA and psoriasis.

RA entails a twofold increase in lymphoma risk and an increased risk for lung cancer. Anti-TNF treatment of RA does not increase overall cancer risk, but increases the risk of NMSC, especially when combined with methotrexate.

In IBD, overall cancer risk is elevated in CD but not in UC. IBD patients run an increased risk for gastrointestinal malignancies driven by chronic intestinal inflammation. Thiopurines increase the risk for NMSC, while limited data are available on the effect of anti-TNF treatment on cancer risk in IBD.

Psoriasis increases the risk for some cancers, such as NMSC and alcohol or smoking-related tumors. Higher cancer risks in patients with severe psoriasis may be confounded by the effect of systemic treatment. PUVA, cyclosporine and anti-TNFs increase NMSC risk in psoriasis patients.

Data on the safety of using biologic or immunosuppressant therapy in IMID patients with a history of cancer are scarce.

## Abbreviations

CD: Crohn’s disease; IBD: Inflammatory bowel disease; IL: Interleukin; IMID: Immune-mediated inflammatory disease; MHC: Major Histocompatibility complex; NMSC: Non-melanoma skin cancer; PUVA: Psoralen – Ultraviolet A; RA: Rheumatoid arthritis; SLE: Systemic Lupus erythematosus; TNF: Tumor necrosis factor; UC: Ulcerative colitis.

## Competing interests

Laurent Beaugerie served as a speaker for Abbott, Ferring and Merck, served as consultant for Abbott.

Lieve Brochez served as speaker for Abbott and Pfizer.

Manuelle Viguier served as speaker for Abbott, Pfizer and Janssen-Cilag.

P Masson served as speaker for Abbott.

E Louis received research support from Abbott, MSD/Schering Plough, AstraZeneca; served as speaker for Abbott, AstraZeneca, Ferring, Falk, MSD/Schering Plough, Menarini, Movetis, Nycomed; and served as consultant for Abbott, AstraZeneca, MSD/Schering Plough, Millenium, Ferring, Shire.

F De Keyser received research support from Roche, UCB, Actelion; served as consultant for MSD/Schering Plough, Abbott, UCB, Roche.

## Authors’ contributions

FDK, EL and PM organized the AIC Spring Lectures and took the initiative to turn their content into a review article. All authors participated in designing the content and setup of the manuscript. R.B performed literature search and data retrieval on the relationship between inflammation and cancer. FDK created the content of the risk of cancer in RA; LB, GVA and EL were responsible for the part on IBD and cancer, MV and LB reviewed the literature on psoriasis and cancer. JCR, VC, GJ, JPM, HP contributed the oncology expert point of view on cancer risk in IMID patients and reviewed the literature on risk of biological treatment in patients with a history of cancer. All authors read and approved the final manuscript.
